# Systemic Effects by Intrathecal Administration of Triamcinolone Acetonide in Patients With Multiple Sclerosis

**DOI:** 10.3389/fendo.2020.00574

**Published:** 2020-08-27

**Authors:** Andreas Hoeflich, Brit Fitzner, Christina Walz, Michael Hecker, Armin Tuchscherer, Manuela Bastian, Julia Brenmoehl, Ina Schröder, Holger S. Willenberg, Martin Reincke, Uwe Klaus Zettl

**Affiliations:** ^1^Institute of Genome Biology, Leibniz Institute for Farm Animal Biology (FBN), Dummerstorf, Germany; ^2^Neuroimmunological Section, Department of Neurology, Rostock University Medical Center, Rostock, Germany; ^3^Institute of Genetics and Biometry, Leibniz Institute for Farm Animal Biology (FBN), Dummerstorf, Germany; ^4^Institute for Clinical Chemistry and Laboratory Medicine, Rostock University Medical Center, Rostock, Germany; ^5^Division of Endocrinology and Metabolism, Rostock University Medical Center, Rostock, Germany; ^6^Department of Endocrinology, Medizinische Klinik und Poliklinik IV, Klinikum der Universität München, München, Germany

**Keywords:** multiple sclerosis, intrathecal administration, cortisol, systemic effects, blood brain-barrier

## Abstract

In patients suffering from multiple sclerosis (MS), intrathecal injection of triamcinolone acetonide (TCA) has been shown to improve symptoms of spasticity. Although repeated intrathecal injection of TCA has been used in a number of studies in late-stage MS patients with spinal cord involvement, no clinical-chemical data are available on the distribution of TCA in cerebrospinal fluid (CSF) or serum. Moreover, the effects of intrathecal TCA administration on the concentrations of endogenous steroids remain poorly understood. Therefore, we have quantified TCA and selected endogenous steroids in CSF and serum of TCA-treated MS patients suffering from spasticity. Concentrations of steroids were quantified by LC-MS, ELISA, or ECLIA and compared with the blood-brain barrier status, diagnosed with the Reibergram. The concentration of TCA in CSF significantly increased during each treatment cycle up to >5 μg/ml both in male and female patients (*p* < 0.001). Repeated TCA administration also evoked serum concentrations of TCA up to >30 ng/ml (*p* < 0.001) and severely depressed serum levels of cortisol and corticosterone (*p* < 0.001). In addition, concentrations of circulating estrogen were significantly suppressed (*p* < 0.001). Due to the potent suppressive effects of TCA on steroid hormone concentrations both in the brain and in the periphery, we recommend careful surveillance of adrenal function following repeated intrathecal TCA injections in MS patients.

## Introduction

Multiple sclerosis (MS) is a chronic inflammatory disease of the central nervous system (CNS) characterized by focal demyelinating lesions, axonal damage, and synaptic loss (1, 2). Immunomodulatory therapies are the primary approach to reduce disease activity. Although the cause of the disease is unknown, a number of genetic (3) and environmental (4) factors are discussed that increase the risk of developing MS. MS is a very heterogeneous disease, and impairment of sensory, cognitive, motor, and/or visual functions can occur during the course of the disease (5). Late-stage patients often show symptoms such as depression, fatigue, paresis, and spasticity. Spasticity severely impairs the patient's abilities and is associated with pain and contractures (6). Treatment options for the medical management of MS-related spasticity include oral application of baclofen (gamma-aminobutyric acid agonist), tizanidine (centrally acting α2 adrenergic agonist), dantrolene sodium (postsynaptic muscle relaxant), and naltrexone (hydrochloride salt) (6, 7). Intrathecal injections of delayed-release steroids such as triamcinolone acetonide (TCA) are a therapeutic option for MS patients with predominantly spinal cord symptoms such as spasticity (8).

TCA belongs to the group of synthetic steroids and represents a corticosteroid with agonistic potential toward the glucocorticoid receptor (GR). The structure of the GR-TCA interaction was resolved recently (9). Intrathecal injection of TCA has been demonstrated to significantly improve spasticity, walking distance, fatigue, and disability in MS patients with otherwise therapy-resistant spasticity (10–17). TCA treatment was also associated with bladder function improvement and increase in quality of life (16). In general, patients with more severe spasticity and higher disability were found to have better clinical outcomes (16). Moreover, a negative correlation between the degree of upper spinal cord atrophy and treatment benefits was shown (18). Despite the invasiveness of the drug delivery, which in some cases may lead to lumbar puncture headache and back pain, intrathecal TCA applications are usually well-tolerated (14, 16). Because there is no long-term improvement of spasticity, repeated cycles of intrathecal TCA injections at intervals of ~3 months are recommended (16).

In cerebrospinal fluid (CSF), repeated intrathecal injection of TCA reduced concentrations of repulsive guidance molecule A (RGMa), an established inhibitor for the regeneration of neurons in the brain (19). The decrease of RGMa levels in CSF was found to be associated with the clinical benefit of TCA treatment in progressive MS patients (20). Studies in mice and rats revealed that RGMa inhibition improves functional recovery by promoting axonal growth and by suppressing inflammation, demyelination, and neurodegeneration (21, 22). It has thus been argued that TCA may not only mediate antispastic effects but also regenerative processes. Although TCA has been used in several studies in MS patients, there is still limited data on whether and to which extent TCA enters the blood from CSF. In addition, the systemic effects of repeated intrathecal TCA injections on the hypothalamic-pituitary-adrenal (HPA) axis remain to be explored in more detail. The present study assessed concentrations of TCA and endogenous steroid hormones in CSF and serum of male and female MS patients with spasticity treated with TCA.

## Methods

### Patients

We used paired CSF and serum samples collected from MS patients with spasticity as part of the administration and monitoring of TCA therapy between 8 a.m. and 11 a.m. The patients gave their prior consent to the use of residual clinical samples for research purposes. The ethics committee of the University Medical Center Rostock approved the use of the samples for this study (approval A 2016-0088). The samples were collected in the years 2009–2012 and stored at−80°C until use. Only complete sample series of patients with at least four applications of TCA (applied every second day), corresponding to one treatment cycle, were included. The administered doses of TCA were allowed to change within treatment cycles and ranged from 40 to 80 mg, dependent on the individual severity of spasticity and the treating physician's assessment. CSF was always collected immediately before intrathecal TCA injection and blood was taken after lumbar puncture. Before assessment of the respective treatment cycles, each patient had received at least one treatment cycle of intrathecal TCA injections. The previous treatment cycle ended around 3 months before. None of the patients has ever been treated with TCA through other routes of administration. We analyzed samples of six treatment cycles from two postmenopausal female patients and of 14 treatment cycles from four male patients (Table 1). Each cycle included four intrathecal TCA injections yielding a total of 80 CSF samples and 80 serum samples. The status of blood-brain barrier was diagnosed with the help of a Reibergram (23).

**Table 1:** Clinicodemographic and pharmacological data.

Cycles per gender, *N* (%)	
Female	6 (30.0)
Male	14 (70.0)
Age in years, mean ± SD	52.9 ± 9.5
Clinical subtype of MS, *N* (%)	
RRMS	2 (10.0)
SPMS	10 (50.0)
PPMS	8 (40.0)
Disease duration in years, median (range)	10 (4–26)
EDSS score, mean ± SD	6.2 ± 1.1
TCA treatment cycle, median (range)	8 (2–21)
TCA cumulative dose in mg, median (range)	240 (160–320)
BBB dysfunction, *N* (%)	
None	11 (55.0)
Slight	6 (30.0)
Moderate	3 (15.0)
Intrathecal IgG synthesis, *N* (%)	
Yes	0 (0.0)
No	20 (100.0)

*Longitudinal sample series (n = 20) were obtained in the course of cyclic intrathecal applications of TCA. In addition to clinicodemographic information, the table provides the patients' total number of treatment cycles received (including the current one), the cumulative dose of TCA administered during the current treatment cycle as well as CSF findings (BBB dysfunction and intrathecal IgG synthesis) at treatment cycle initiation. BBB, blood-brain barrier; CSF, cerebrospinal fluid; EDSS, expanded disability status scale; IgG, immunoglobulin G; MS, multiple sclerosis; N, number; PPMS, primary progressive multiple sclerosis; RRMS, relapsing-remitting multiple sclerosis; SD, standard deviation; SPMS, secondary progressive multiple sclerosis; TCA, triamcinolone acetate*.

### Steroid Analysis by Mass Spectrometry

Steroid profiles were quantified by liquid chromatography coupled mass spectrometry (LC-MS/MS) as describe before by Kunze et al. (24). Serum and CSF were precipitated in methanol/acetonitrile/acetone (1:1:1) by sonication and the precipitate was removed by centrifugation. Dried supernatants were stored at −20°C. For LC-MS/MS analysis, samples were dissolved in 50% methanol containing 100 ng/ml internal standard (dexamethasone). LC-MS analysis for steroids was performed using an Accela HPLC system (Thermo Fisher Scientific, Dreieich, Germany) with Accucore C18 column (2.6 μm × 50 mm × 2.1 mm) coupled to the LTQ Orbitrap high-resolution hybrid mass spectrometer (Thermo Fisher Scientific) using ESI source (24). For each analyte, a calibrator set of nine concentrations (ranging between 0 and 500 ng/ml) was added to an analyte-free matrix using dexamethasone as an internal standard. Curve fitting was performed by non-linear regression (third-order polynomial function). The lower limit of quantification (LLOQ) was determined for estriol: 21 ng/ml; cortisol: 2 ng/ml; 11-ketotestosterone: 1 ng/ml; androstenedione: 1 ng/ml; 4-pregnen-17α,20β-diol-3-one: 2.5 ng/ml; TCA: 1 ng/ml; corticosterone: 1 ng/ml; and progesterone: 1 ng/ml.

### Quantification of 17 β-Estradiol and Testosterone in Serum by ELISA and ECLIA

17 β-estradiol could not be determined by electrospray ionization and was therefore quantified in serum only with a highly sensitive ELISA kit according to the instructions of the manufacturer (ADI-901-174, Enzo Life Sciences Inc.; Lörrach, Germany). The sensitivity or limit of detection (LOD) of this assay is 14 pg/ml. For precipitation, a cold mixture of acetone, acetonitrile, and methanol (1:1:1) was added to serum (8:1). After centrifugation, supernatants were dried and used for rehydration in kit assay buffer. LC-MS could also not be used for the quantification of testosterone because the peak was overlaid by an unknown metabolite characterized by the identical mass-to-charge ratio compared to testosterone standard. Therefore, testosterone was quantified in serum using the Elecsys Testosterone II ECLIA kit on a cobas e 411 according to the manufacturer's instructions (05200067 190, Roche Diagnostics GmbH, Mannheim, Germany). The limit of detection of this assay is 0.025 ng/ml.

### Statistical Analyses

The data analysis was generated using SAS software, Version 9.4 for Windows (SAS Institute Inc., Cary, NC, USA). Descriptive statistics and tests for normality were performed by use of the UNIVARIATE procedure of Base SAS software. CSF and serum data were normally distributed and were assessed by repeated measurement analyses of variance using the MIXED procedure included in the SAS/STAT software package. The repeated measurement ANOVA model for CSF and serum data contained the fixed factors gender (levels: female, male), application (levels: I, II, III, IV), the interaction gender^*^application and the covariate treatment cycle. An extended model was derived by additionally including the factor administered TCA dose (levels: 40, 60, 80 mg). Repeated measures on the same patient (treatment cycle, application) were considered by the REPEATED statement of the MIXED procedure using the SUBJECT = patient option for the definition of the blocks from the block-diagonal residual covariance matrix and the TYPE = CS option in order to define their covariance structure.

Another repeated measurement ANOVA model for TCA in CSF and serum contained the fixed factors gender (levels: female, male), application (levels: I, II, III, IV), blood-brain barrier (BBB) dysfunction [levels: none, slight, moderate (25, 26)], the interactions gender^*^application, gender^*^BBB dysfunction and the covariate treatment cycle. Repeated measures on the same patient were handled in the same way as described above.

Least-squares means (LS-means) and their standard errors (SE) were calclulated for each fixed effect in the models, and the pairwise differences of all LS-means were tested by the Tukey-Kramer test. Partitioned analyses of the LS-means were performed by use of the SLCE statement of the MIXED procedure for the two-way interaction gender^*^application (i.e., test of the gender within the levels of application and test of the application within the levels of gender) and for the interaction gender^*^BBB dysfunction (i.e., test of the gender within the levels of BBB dysfunction and test of BBB dysfunction within the levels of gender).

The Pearson correlation of TCA concentrations present in CSF and serum of MS patients after repeated intrathecal TCA injections was estimated and tested with the CORR procedure of Base SAS software.

Test results were considered significant if *p* < 0.05.

Figures were created with SigmaPlot for Windows Version 14.0 and JMP Version 13.1.0 for Windows (SAS Institute Inc., Cary, NC, USA).

## Results

Patients suffering from MS with spasticity were treated with repeated intrathecal injections of TCA. A total of 20 treatment cycles in 6 patients (2 females and 4 males), who already received at least one previous treatment cycle, were analyzed in this study. Each treatment cycle consisted of 4 consecutive TCA applications in 2-day intervals. Clinical and demographical data of the patients are summarized in Table 1. For each patient, also an individual data set is provided by Supplementary Tables 1A,B. further containing the concentrations of steriods in serum and CSF. Spasticity improved in all patients due to TCA treatment.

### Concentrations of TCA in CSF and Serum

**Figure 1:**
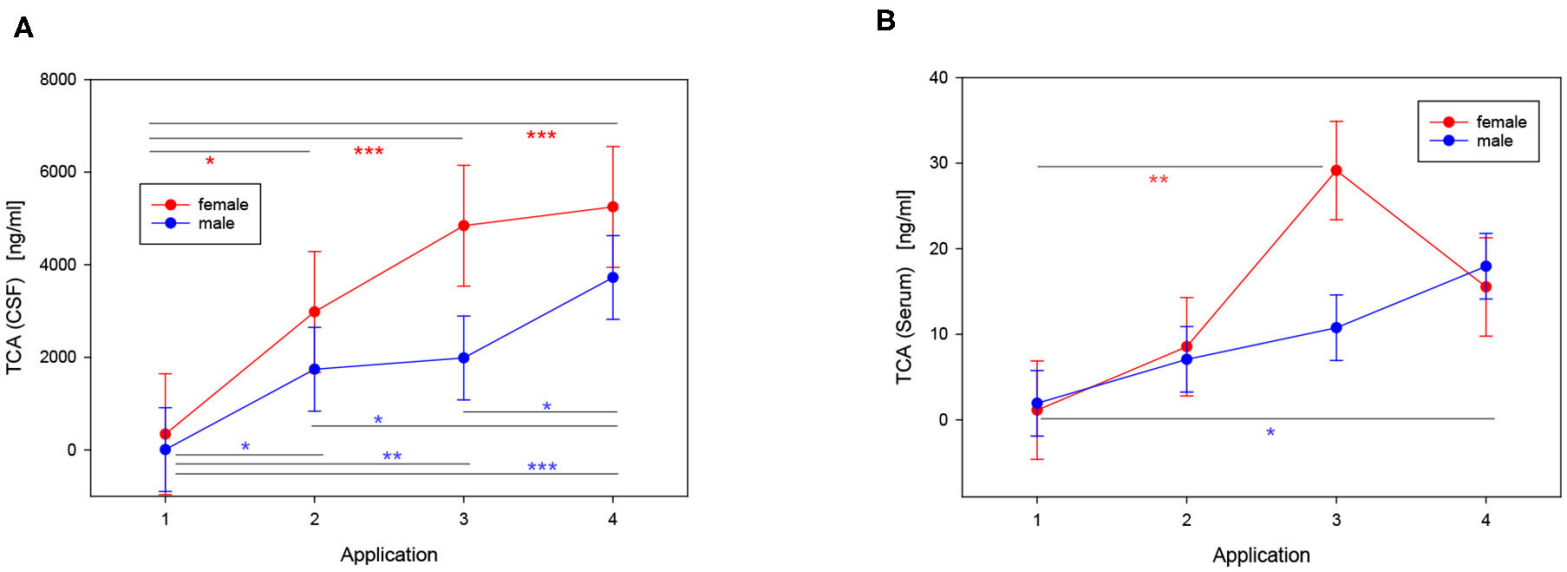
Effects of repeated intrathecal TCA injections in male (blue) and female (red) MS patients on TCA concentrations in CSF **(A)** and serum **(B)** (LS-means ± SE). The injections were performed in treatment cycles consisting of 4 consecutive applications with 2-day intervals. The peak in **(B)** is driven by an outlier in the data Supplementary Table 1 that presumably resulted from a traumatic lumbar puncture (pairwise multiple comparisons with the Tukey-Kramer procedure: *p < 0.05; **p < 0.01; ***p < 0.001; n_cycles_ = 20: 6 cycles of 2 females, 14 cycles of 4 males).

**Figure 2:**
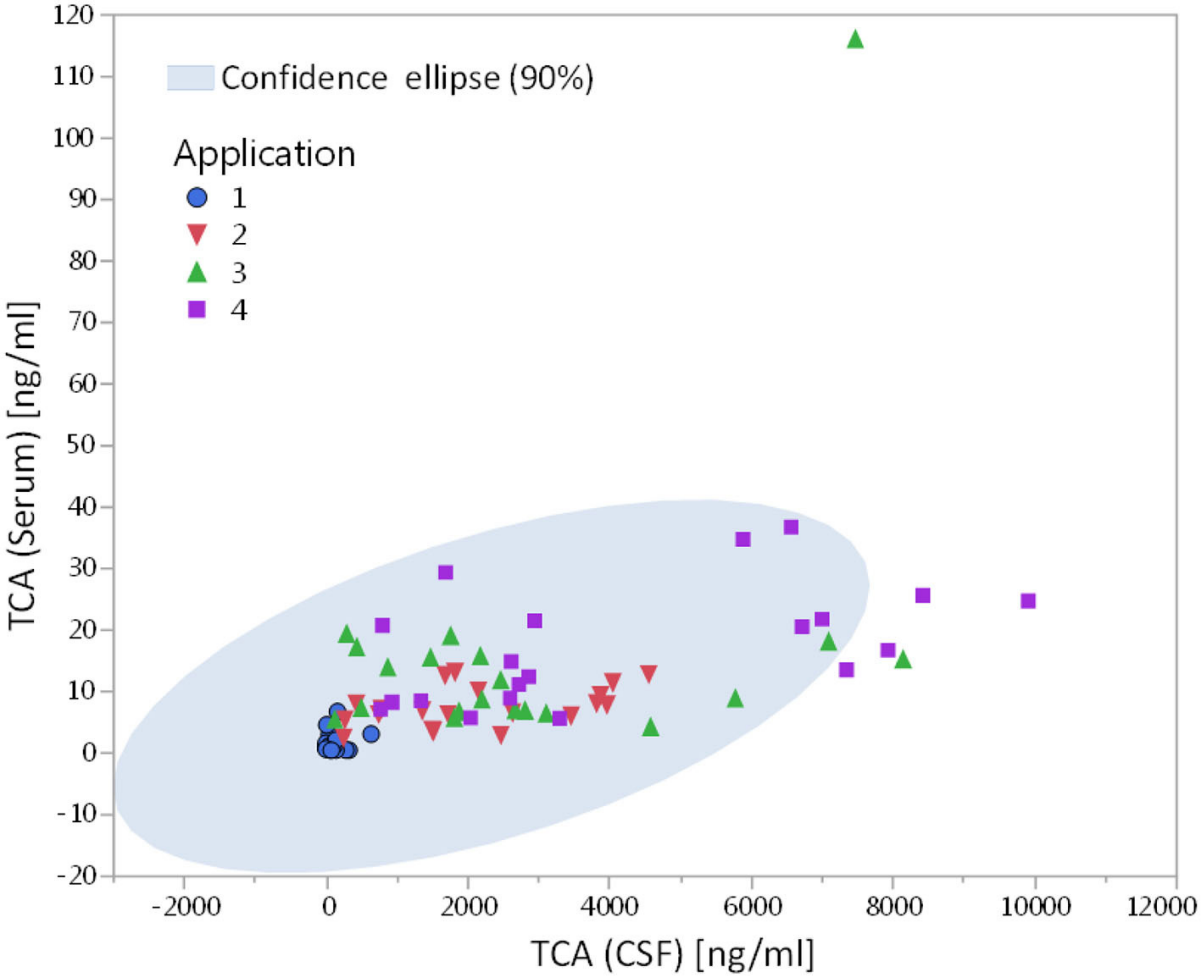
Correlation of TCA concentrations present in CSF and serum in MS patients receiving repeated intrathecal TCA injections (Pearson correlation coefficient r = 0.541, *p* < 0.001; n_cycles_ = 20: 6 cycles of 2 females, 14 cycles of 4 males).

TCA concentrations in CSF and serum were quantified by LC-MS (Figure 1A). At the time point of the initial injection, TCA was detectable in 15 out of 20 CSF samples, with concentrations ranging up to 636 ng/ml. Compared to the first application time point, TCA concentrations were significantly increased (*p* < 0.001) at application 2, 3, and 4, even up to 30-fold at the end of the treatment cycle (LS-means: TCA_application1_: 151 ng/ml, TCA_application2_: 2,362 ng/ml, TCA_application3_: 3,416 ng/ml, TCA_application4_: 4,488 ng/ml; SE: 794). In order to estimate the potential transfer of TCA during or after lumbar puncture to the circulation, corresponding serum samples were analyzed as well (Figure 1B). In contrast to CSF, at the time of the first injection, serum TCA concentrations were close to the lower limit of quantification. However, similar to CSF, albeit at much lower levels, the concentrations of TCA in serum were increased by an order of magnitude at the end of the treatment cycle compared to application 1 (LS-means: TCA_application1_: 1.5 ng/ml, TCA_application2_: 7.8 ng/ml, TCA_application3_: 20.0 ng/ml, TCA_application4_: 16.7 ng/ml; SE: 3.4). Stratified by gender, the concentrations of TCA in serum were significantly different between application 1 and 3 in female patients (*p* < 0.01) and between application 1 and 4 in male patients (*p* < 0.05). The effect of the individual cumulative dosage administered on TCA concentrations quantified in CSF and serum is visualized in Supplementary Figure 1. The extended models revealed that the measured levels of TCA in both CSF and serum were associated with the individual doses of TCA injected in the course of each cycle (*p* < 0.05). We found no significant drug dose dependency for the other steroid concentrations, except for 11-ketotestosterone levels in CSF, which were negatively associated with TCA dosage (*p* = 0.008). Moreover, TCA concentrations in CSF significantly correlated with TCA levels in serum (Figure 2; Pearson correlation: 0.541, *p* < 0.001).

### Effects of Intrathecal TCA Injection on Endogenous Steroid Hormones in Serum

**Figure 3:**
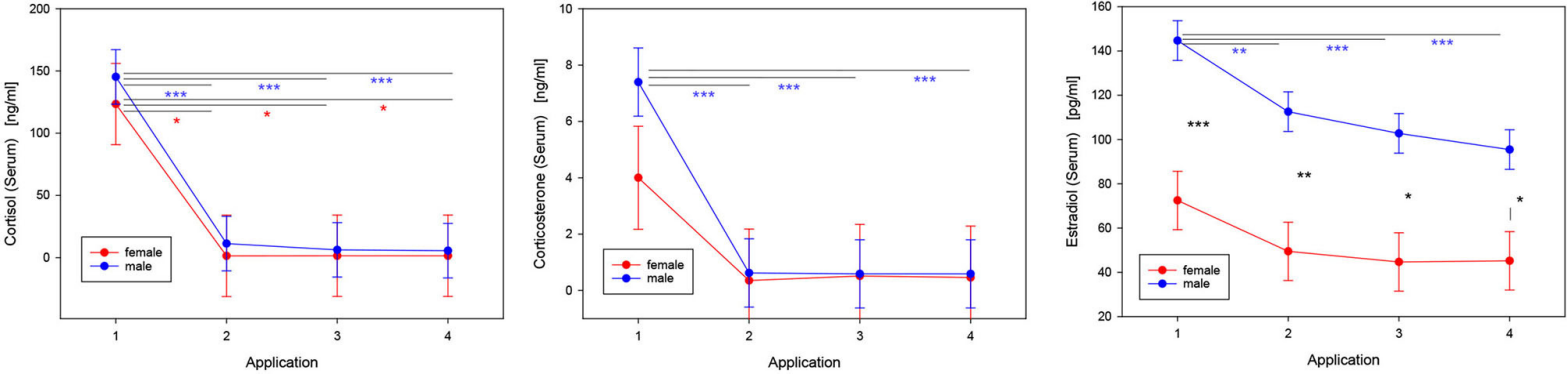
Effects of repeated intrathecal TCA administration on concentrations of steroid hormones in serum. LS-means and SE are shown for those steroids that were significantly modulated in response to the therapy (Table 2). The injections of TCA were performed in treatment cycles consisting of 4 consecutive applications with 2-day intervals (pairwise multiple comparisons with the Tukey-Kramer procedure: *p < 0.05; **p < 0.01; ***p < 0.001; n_cycles_ = 20: 6 cycles of 2 females, 14 cycles of 4 males).

Intrathecal injection of TCA efficiently suppressed circulating concentrations of different steroids (Table 2, Figure 3). In male patients, the serum concentrations of cortisol, corticosterone, and E2 were significantly decreased as early as 2 days after the first injection of TCA (*p* < 0.01). The TCA injections had a similarly strong effect on the concentrations of cortisol in female patients (*p* < 0.05). Circulating concentrations of estriol in serum were unaffected by TCA injections (Table 2). Also testosterone, quantified by ECLIA in 33 sera from male patients (insufficient sample material resulted in missing data), was significantly reduced in the course of repeated TCA injections (*p* < 0.001). Accordingly, concentrations of testosterone dropped from 3.9 ± 1.1 ng/ml (*n* = 7) at the time point of the first TCA application to 0.9 ± 0.6 ng/ml (*n* = 9) at the time point of the fourth injection.

**Table 2:** Significance values for steroid concentrations in CSF and serum during TCA treatment of MS patients.

	**CSF**			**Serum**		
**Steroid hormone**	**Application**	**Gender**	**Interaction**	**Application**	**Gender**	**Interaction**
TCA	**6.7E-12**	0.371	0.101	**1.0E-04**	0.434	0.054
**Mineralo- and glucocorticoids**						
Corticosterone	0.508	0.305	0.838	**3.6E-04**	0.537	0.564
Cortisol	**5.3E-05**	0.095	0.111	**1.6E-08**	0.692	0.986
**Androgens**						
11-keto-testosterone	0.527	**0.016**	0.527	0.421	0.064	0.421
Androstenedione	0.360	0.445	0.758	0.718	0.666	0.263
**Progestagens**						
17α,20β-DP	–	–	–	0.084	0.646	0.822
Progesterone	0.919	0.167	0.930	0.174	**0.039**	0.375
**Estrogens**						
Estradiol	n.d.	n.d.	n.d.	**1.5E-05**	**0.033**	0.552
Estriol	–	–	–	0.468	0.501	0.723

*The table provides p-values for the main and interaction effects of drug application and gender as calculated using linear models adjusted for treatment cycle. Statistically significant effects (p < 0.05) are indicated in bold. –, not detected; 17α,20β-DP, 17α,20β-dihydroxy-4-pregnen-3-one; CSF, cerebrospinal fluid; MS, multiple sclerosis; n.d., not determined; TCA, triamcinolone acetate*.

### Concentrations of Steroids in CSF

In CSF, at the time of the first TCA injection, the mean concentration of cortisol was 9.2 ± 1.3 ng/ml. At all the following application time points, cortisol was undetectable in most of the patients' CSF samples (*p* < 0.001; Table 2). The levels of the analyzed endogenous steroid hormones were generally very low in CSF. The full data set is provided in Supplementary Table 1.

### Effect of Gender

In CSF, males had higher concentrations of 11-ketotestosterone compared to females (males: 0.66 ± 0.03 ng/ml; females: 0.48 ± 0.04 ng/ml; *p* < 0.05). In serum, concentrations of E2 were generally higher in male compared to female postmenopausal patients (*p* < 0.05) (Figure 3). In contrast, progesterone levels in serum were significantly higher (*p* < 0.05) in females (0.55 ± 0.01 ng/ml) compared to males (0.52 ± 0.005 ng/ml). In CSF, concentrations of cortisol were, on average, significantly higher in females (13.3 ± 2.2 ng/ml) than in males (5.0 ± 1.4 ng/ml) at the time of the first application (*p* < 0.05). All other steroids measured were unaffected by gender.

### Effects of Blood-Brain Barrier (BBB) Dysfunction on TCA Concentrations in Serum and CSF

We hypothesized, that TCA concentrations in serum might be affected by BBB disruption. Accordingly, potential effects of BBB dysfunction were assessed in a separate statistical model including gender and BBB dysfunction categorized in 3 distinct classes (moderate, slight, and no dysfunction). Functionality of the BBB had no effect (*p* = 0.798) on concentrations of TCA in serum Supplementary Figure 2. However, moderate dysfunction of the BBB was associated with higher (*p* < 0.01) concentrations of TCA in CSF in the joint collective of male and female patients (no dysfunction: 1,688 ± 354 ng/ml; slight dysfunction: 2,165 ± 452 ng/ml; moderate dysfunction: 3,640 ± 472 ng/ml). Immediately before the first intrathecal injections of TCA, the ratios of corticosterone/cortisol in CSF and serum were 0.05 and 0.1, respectively.

## Discussion

TCA as a synthetic glucocorticoid has strong anti-inflammatory and immuno-suppressive potential as well as regenerative properties. It is approved in clinical settings, e.g., rheumatoid and allergic diseases. By contrast, MS represents an unapproved indication for the application of TCA and, thus, TCA is used *off-label* in selected MS patients with predominant spinal cord symptoms such as intractable spasticity (10–14). Our study demonstrates effects of repeated intrathecal TCA injections on steroid concentrations in CSF and serum from male and female late-stage MS patients suffering from spasticity.

Repeated intrathecal injections of TCA substantially increased concentrations of TCA in CSF toward the end of the treatment cycles. Also in serum, significant increases of TCA were quantified in the course of each cycle although in serum, the concentrations of TCA were 2–3 orders of magnitude lower as compared to CSF. Rohrbach et al. (27) had assessed TCA concentrations in the CSF after repeated intrathecal TCA injections. Although we do not have available data on distinct TCA concentrations from this mentioned study, TCA was detectable in the CSF 4–6 months after injection in some of the patients, suggesting longer lasting effects of intrathecal vs. oral application. In our study, TCA was detected in most of the CSF samples (15/20) immediately before the first intrathecal injection, which supports carry over of TCA from the previous treatment cycle (≈3 months before) and thus long-lasting effects of intrathecal TCA injections. Furthermore, also in serum, considerable levels of TCA were quantified at the time point of the first application, which also might originate from the previous cycle of injections or from traumatic intrathecal injection at the time of application. From this finding and due to the correlation between the concentrations of TCA in serum and CSF, concentrations of serum TCA levels could be considered as markers for the concentrations of TCA in the central nervous system (CNS) in MS-related spasticity. In conjunction with other clinical parameters, serum TCA levels might be useful in order to adapt individual treatment dosage or lengths of interval between TCA treatment cycles.

### Effects of the Blood-Brain Barrier (BBB) on the Distribution of TCA in CSF and Serum

In general, the much higher concentrations of TCA in CSF than in serum in all patients regardless of the condition of the BBB indicate that the BBB is an efficient barrier against free distribution of TCA in different compartments in all patients. However, in patients with moderate BBB dysfunction, the TCA concentrations in CSF were higher if compared to other patients. In the presence of an intact BBB, TCA thus appears to be released from CSF to the circulation. Because oral application of TCA resulted in low levels of TCA in the CSF (27), we may assume bidirectional although restricted permeability of the BBB for the TCA molecule. It is well-known that a number of adrenal and gonadal steroids can cross the BBB (28) and the transfer of steroids from the circulation to the CNS were originally thought to represent a function of steroid concentrations (29). However, transport of free steroids through membranes appeared to depend on the interactions of the steroid with water molecules by means of hydrogen bonds (28). In addition, transfer of TCA through the BBB is restricted by P-glycoprotein (P-gp) transporters (30) belonging to the family of ATB binding cassette transporters, which are involved in the bidirectional transport e.g., of sulfated steroids through the BBB (31). Although the transport efficiency of P-gp for TCA is much lower compared to dexamethasone (32), the lack of P-gp in mice resulted in higher levels of TCA in their CNS (30). Accordingly, the increased TCA concentrations in the CSF in MS patients with moderate BBB dysfunction could be related to the P-gp transporters. Due to the selective transport of different steroids by P-gp, different ratios e.g., of corticosterone/cortisol in plasma vs. CNS extracts were explained (33). Notably, in our study we were able to confirm the ratio of both hormones in the circulation (corticosterone/cortisol: ≈0.05) described before in human plasma (33). In human CNS extracts the mentioned ratio was around 0.3 (33), which is higher than the CSF ratio of both steroids measured in our study (0.1). The differences may be due to the different specimens or to the different functionalities of the BBB in both studies.

### Effects of Intrathecal TCA Injection on Concentrations of Endogenous Steroid Hormones

Very clearly, repeated injections of TCA suppressed cortisol, corticosterone and estradiol concentrations in serum. It would be interesting to evaluate in larger studies whether the TCA-induced steroid dynamics in serum correlate with the patients' heterogeneous clinical response to therapy. It is important to note, that the concentrations of glucocorticoids were not elevated at the start of each cycle, e.g., in response to stress, and then reduced toward later stages due to habituation. Instead, the concentrations of both glucocorticoids started from normal levels (34) and were reduced to almost undetectable levels during the treatment cycles. Also in CSF, cortisol was markedly suppressed in response to TCA injection. The negative effect of intrathecal TCA injection on cortisol production has been reported previously (27, 35). More specifically, Neu et al. observed an almost 50% decrease in endogenous cortisol levels within 3 days following a single intrathecal dose of 40 mg TCA (35). In comparison to their study, the higher injected cumulative dosage of TCA in our study presumably led to an even more pronounced reduction in serum cortisol levels. It is commonly accepted that systemic or local application of TCA in various diseases may result in suppression of the hypothalamic-pituitary-adrenal axis and development of the Cushing's syndrome (36, 37). One of the principal reasons for the intrathecal administration of TCA was to avoid the systemic effects of TCA. This argument is not strictly supported by the data presented in this manuscript, although the concentrations of TCA in the circulation were much lower compared to those in CSF and no systemic side effects were clinically evident. Nevertheless, monitoring endogenous secretion of cortisol in response to intrathecal TCA treatment warrants consideration in subsequent observational studies.

We are well aware that this retrospective study has clear limitations. Due to the fact that this is an initial report and sample volumes were limited, it was impossible to quantify additional hormones such as ACTH, LH, FSH, or renin. Furthermore, given the severity of the disease it was impossible so far to perform functional diagnostics, such as ACTH stimulation, in the MS patients. The present study only included a small number of individual patients and was further characterized by gender imbalance. Therefore, and with respect to the heterogeneous therapeutic drug response in humans in general it is not possible to superimpose the effects of repeated intrathecal TCA injection described here to a broader human population. Finally, the retrospective study included patients that had obtained different TCA doses, which may have different effects on the steroid concentrations measured.

To summarize, repeated intrathecal injections of TCA in MS patients resulted in substantial gradual increases of TCA concentrations both in CSF and serum. TCA concentrations in CSF and serum correlated significantly, and we may consider the use of serum TCA concentrations as a marker that complements the clinical aspects of care to fine-tune the therapy of MS-related spasticity in individual patients. Very clearly, intrathecal injections of TCA robustly suppress the HPA axis suggesting careful surveillance of adrenal functions in these patients.

## Data Availability Statement

All datasets presented in this study are included in the article/Supplementary Material.

## Ethics Statement

The use of samples from human participants for this study was reviewed and approved by the Ethics Committee of the University Medical Center Rostock (approval A 2016-0088). The patients gave their prior consent to the use of residual clinical samples for research purposes.

## Author Contributions

All authors wrote and approved the manuscript. CW and MB performed the analysis. AT performed the statistical analysis. UZ performed the clinical study.

## Conflict of Interest

The authors declare that the research was conducted in the absence of any commercial or financial relationships that could be construed as a potential conflict of interest.
