# Supplementary material for: Systemic Effects by Intrathecal Administration of Triamcinolone Acetonide in Patients With Multiple Sclerosis
Source: Front Endocrinol (Lausanne). 2020 Aug 27;11:574. doi: 10.3389/fendo.2020.00574 (PMC7481359; doi:10.3389/fendo.2020.00574)
Supplement: Supplementary file 1 [file Data_Sheet_1.pdf]

Suppl. Figure 1

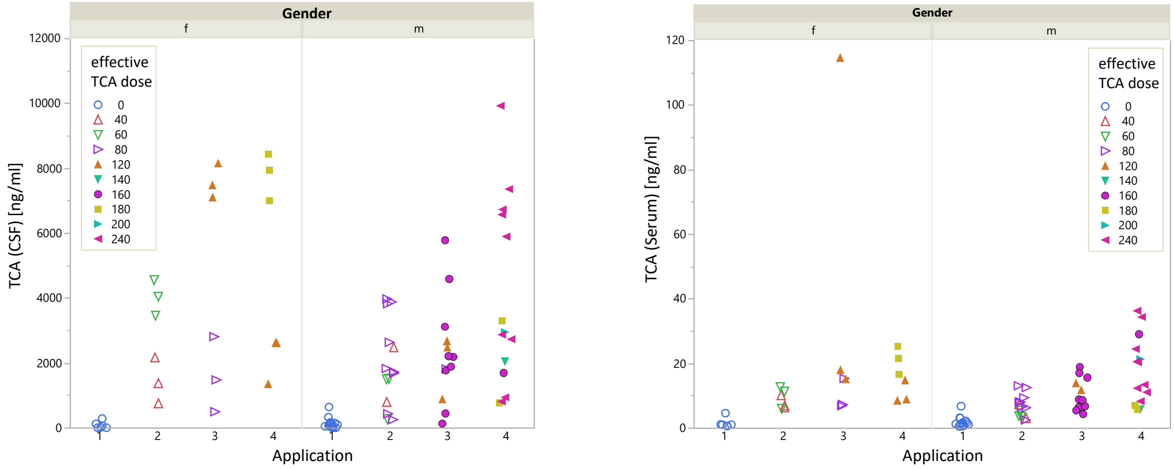

Concentrations of TCA in CSF and serum of female and male MS patients, with the colors and shapes of the markers indicating the individual cumulative dose of TCA received during the current treatment cycle, which consisted of 4 consecutive intrathecal injections of TCA in 2-day intervals (n_{cycles}=20: 6 cycles of 2 females, 14 cycles of 4 males).

Suppl. Figure 2

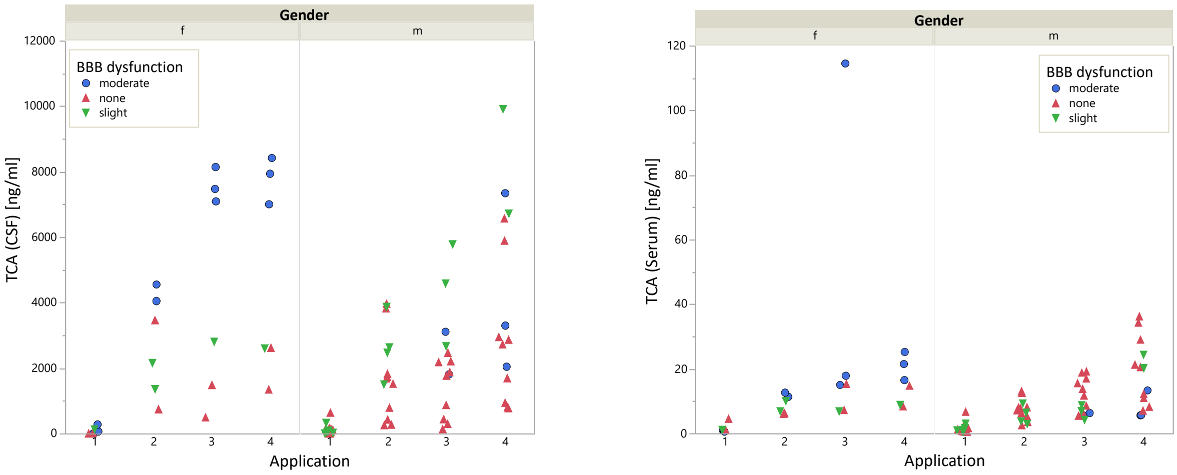

Concentrations of TCA in CSF and serum of MS patients receiving repeated intrathecal TCA injections, visualizing the individual degree of BBB disruption at each time point. TCA was administered in treatment cycles consisting of 4 consecutive applications with 2-day intervals (n_{cycles}=20: 6 cycles of 2 females, 14 cycles of 4 males).

Suppl. Table 1A Steroid concentrations in serum from MS patients after intrathecal TCA administration

Patient	TCA treat-ment cycle	Intra-thecal appli-cation	TCA dose [mg]	Gender	Clinical subtype of MS	BBB dysfu-nction	Intrathecal IgG synthesis	TCA in serum [ng/ml]	Cortico-sterone in serum [ng/ml]	Cortisol in serum [ng/ml]	11-Ketotesto-sterone in serum [ng/ml]	Andro-stendione in serum [ng/ml]	Testo-sterone in serum [ng/ml]	17α,20β-DP in serum [ng/ml]	Proge-sterone in serum [ng/ml]	Estradiol in serum [pg/ml]	Estriol in serum [ng/ml]
Pat1	18	1	80	Male	PPMS	Slight	No	1.64	12.99	204.21	1.60	0.93	4.85	2.03	0.55	192.06	30.70
Pat1	18	2	80	Male	PPMS	None	Yes	12.51	0.50	0.96	0.73	0.50	1.56	1.25	0.62	137.76	30.14
Pat1	18	3	80	Male	PPMS	None	Yes	18.81	0.50	0.96	0.91	0.50	1.37	1.25	0.56	117.63	10.41
Pat1	18	4	80	Male	PPMS	None	Yes	36.24	0.50	0.96	0.60	0.77	1.12	1.25	0.55	82.32	23.40
Pat1	19	1	80	Male	PPMS	Slight	No	1.00	11.63	200.19	0.79	0.77	5.28	2.42	0.54	179.75	26.58
Pat1	19	2	80	Male	PPMS	Slight	Yes	9.32	0.50	0.96	0.55	0.50	2.90	1.25	0.51	130.72	20.89
Pat1	19	3	80	Male	PPMS	Moderate	No	6.35	0.50	0.96	0.58	0.77	2.44	1.29	0.54	100.15	37.12
Pat1	19	4	80	Male	PPMS	Slight	Yes	20.29	0.50	0.96	0.63	0.50	0.69	1.25	0.51	81.55	15.06
Pat1	20	1	80	Male	PPMS	Slight	No	1.04	10.99	252.48	1.28	0.86		2.11	0.58	163.01	10.41
Pat1	20	2	80	Male	PPMS	None	Yes	8.05	0.50	0.96	0.50	0.50	2.26	1.25	0.57	106.80	10.41
Pat1	20	3	80	Male	PPMS	Slight	No	8.80	0.50	0.96	1.04	0.86	1.41	1.25	0.51	106.68	10.41
Pat1	20	4	80	Male	PPMS	Slight	No	24.39	0.50	0.96	1.11	0.50	0.46	1.25	0.58	99.06	10.41
Pat1	21	1	80	Male	PPMS	Slight	No	3.02	5.94	199.33	2.12	0.50	4.44	1.25	0.59	164.20	10.41
Pat1	21	2	80	Male	PPMS	Slight	Yes	6.51	0.50	22.17	0.97	0.85	2.38	1.25	0.57	151.69	10.41
Pat1	21	3	80	Male	PPMS	Slight	No	4.22	0.50	0.96	0.98	0.50	1.76	1.25	0.51	120.71	10.41
Pat1	21	4	40	Male	PPMS	Moderate	No	13.35	0.50	0.96	1.00	0.50	1.55	1.25	0.51	80.50	10.41
Pat2	8	1	40	Female	SPMS	None	No	1.01	0.50	257.32	0.50	0.50		1.25	0.54	77.27	10.41
Pat2	8	2	40	Female	SPMS	Slight	No	6.87	0.50	0.96	0.50	0.50		1.25	0.58	60.31	27.91
Pat2	8	3	40	Female	SPMS	None	No	7.25	1.24	0.96	0.50	0.50		1.25	0.51	47.51	24.82
Pat2	8	4	40	Female	SPMS	None	No	8.38	1.14	0.96	0.50	0.85		1.25	0.61	38.82	34.71
Pat2	9	1	40	Female	SPMS	None	No	4.56	0.50	0.96	0.50	0.77		1.25	0.54	73.73	46.23
Pat2	9	2	40	Female	SPMS	None	No	6.31	0.50	1.41	0.50	0.79		1.25	0.60	70.10	25.30
Pat2	9	3	40	Female	SPMS	None	Yes	15.32	0.50	0.96	0.50	0.79		1.25	0.58	43.13	20.51
Pat2	9	4	40	Female	SPMS	None	Yes	14.71	0.50	0.96	0.50	0.77		1.25	0.57	61.96	28.63
Pat2	14	1	40	Female	SPMS	Moderate	No	0.98	0.50	0.96	0.50	0.50		1.25	0.51	48.01	10.41
Pat2	14	2	40	Female	SPMS	Slight	No	10.04	0.50	0.96	0.50	0.86		1.25	0.60	35.66	31.78
Pat2	14	3	40	Female	SPMS	Slight	No	6.85	0.73	0.96	0.50	0.50		1.25	0.58	47.51	10.41
Pat2	14	4	40	Female	SPMS	Slight	No	8.83	0.50	0.96	0.50	0.50		1.25	0.59	40.89	10.41

Patient	TCA treat-ment cycle	Intra-thecal appli-cation	TCA dose [mg]	Gender	Clinical subtype of MS	BBB dysfu-nction	Intrathecal IgG synthesis	TCA in serum [ng/ml]	Cortico-sterone in serum [ng/ml]	Cortisol in serum [ng/ml]	11-Ketotesto-sterone in serum [ng/ml]	Andro-stendione in serum [ng/ml]	Testo-sterone in serum [ng/ml]	17α,20β-DP in serum [ng/ml]	Proge-sterone in serum [ng/ml]	Estradiol in serum [pg/ml]	Estriol in serum [ng/ml]
Pat3	4	1	60	Male	SPMS	None	No	6.72	28.65	263.85	2.13	0.83		9.68	0.51	127.62	32.61
Pat3	4	2	60	Male	SPMS	Slight	No	3.80	0.50	14.61	0.88	0.72	1.83	2.02	0.51	98.82	10.41
Pat3	4	3	60	Male	SPMS	Slight	No	6.92	0.50	0.96	0.81	0.50	1.74	2.11	0.51	102.32	10.41
Pat3	4	4	60	Male	SPMS	Moderate	No	5.62	0.50	0.96	0.93	0.50	0.82	1.98	0.51	108.05	29.84
Pat3	5	1	40	Male	SPMS	Slight	No	0.50	0.50	37.34	0.73	0.70		2.25	0.51	97.26	29.54
Pat3	5	2	40	Male	SPMS	Slight	No	2.94	0.50	0.96	1.03	0.70		2.14	0.51	119.25	32.77
Pat3	5	3	60	Male	SPMS	Moderate	No	5.65	0.50	0.96	0.97	0.50		1.25	0.51	75.16	32.72
Pat3	5	4	60	Male	SPMS	Moderate	No	5.71	0.50	0.96	0.50	0.50		1.96	0.51	14.00	10.41
Pat3	10	1	80	Male	SPMS	None	No	1.62	6.47	181.85	1.12	0.88	3.23	1.25	0.58	109.48	10.41
Pat3	10	2	80	Male	SPMS	None	No	7.84	0.50	0.96	1.07	0.50	1.70	1.25	0.51	79.19	10.41
Pat3	10	3	80	Male	SPMS	None	No	6.67	0.50	0.96	0.98	0.50		1.25	0.51	87.59	10.41
Pat3	10	4	40	Male	SPMS	None	No	11.03	0.50	0.96	0.98	0.86	0.46	1.25	0.51	74.92	24.62
Pat3	11	1	60	Male	SPMS	None	No	1.18	1.20	160.08	1.29	0.50	2.63	1.25	0.51	102.36	10.41
Pat3	11	2	60	Male	SPMS	None	No	3.58	0.50	0.96	0.98	0.50	2.20	1.25	0.51	65.07	10.41
Pat3	11	3	80	Male	SPMS	None	No	11.70	0.50	0.96	1.04	0.98	1.67	1.25	0.51	80.78	28.06
Pat3	11	4	40	Male	SPMS	None	Yes	21.27	0.50	0.96	0.50	0.86	0.21	1.25	0.51	151.84	44.72
Pat4	2	1	60	Female	SPMS	Moderate	No	0.60	1.40	142.88	0.50	0.69		2.06	0.51	42.37	10.41
Pat4	2	2	60	Female	SPMS	Moderate	No	11.36	0.50	0.96	0.50	0.50		1.25	0.51	31.60	30.07
Pat4	2	3	60	Female	SPMS	Moderate	No	17.88	0.50	0.96	0.50	0.70		2.16	0.51	43.53	56.11
Pat4	2	4	60	Female	SPMS	Moderate	No	16.52	0.50	0.96	0.50	0.73		1.25	0.51	39.47	51.22
Pat4	3	1	60	Female	SPMS	Moderate	No	0.58	13.62	188.74	0.50	0.50		1.25	0.51	68.08	35.12
Pat4	3	2	60	Female	SPMS	Moderate	No	12.68	0.50	0.96	0.50	0.73		1.25	0.51	14.00	10.41
Pat4	3	3	60	Female	SPMS	Moderate	No	114.54	0.50	0.96	0.50	0.74		1.25	0.51	33.80	40.66
Pat4	3	4	60	Female	SPMS	Moderate	No	21.51	0.50	0.96	0.50	0.76		1.25	0.51	49.73	37.90
Pat4	6	1	60	Female	SPMS	Slight	No	1.12	8.38	164.58	0.50	0.91		14.21	0.60	120.83	25.56
Pat4	6	2	60	Female	SPMS	None	No	6.05	0.50	0.96	0.50	0.89		9.03	0.59	80.59	45.67
Pat4	6	3	60	Female	SPMS	Moderate	No	15.05	0.50	0.96	0.50	0.87		1.25	0.51	48.07	29.78
Pat4	6	4	60	Female	SPMS	Moderate	No	25.28	0.50	0.96	0.50	0.86		5.00	0.51	35.72	27.90

Patient	TCA treat-ment cycle	Intra-thecal appli-cation	TCA dose [mg]	Gender	Clinical subtype of MS	BBB dysfu-nction	Intrathecal IgG synthesis	TCA in serum [ng/ml]	Cortico-sterone in serum [ng/ml]	Cortisol in serum [ng/ml]	11-Ketotesto-sterone in serum [ng/ml]	Andro-stendione in serum [ng/ml]	Testo-sterone in serum [ng/ml]	17α,20β-DP in serum [ng/ml]	Proge-sterone in serum [ng/ml]	Estradiol in serum [pg/ml]	Estriol in serum [ng/ml]
Pat5	5	1	60	Male	PPMS	None	No	0.58	0.50	1.94	0.50	0.50		2.03	0.51	163.38	38.83
Pat5	5	2	60	Male	PPMS	None	No	5.37	0.50	0.96	1.05	0.72		2.04	0.51	115.98	20.54
Pat5	5	3	60	Male	PPMS	None	No	13.78	0.50	0.96	0.80	0.72		1.92	0.51	103.23	10.41
Pat5	5	4	60	Male	PPMS	None	No	7.03	0.50	0.96	1.44	0.72		2.10	0.51	112.07	29.75
Pat5	6	1	80	Male	PPMS	None	No	1.22	0.50	0.96	1.04	0.72		2.02	0.51	141.30	28.76
Pat5	6	2	80	Male	PPMS	None	No	2.48	0.50	0.96	0.93	0.50		1.93	0.51	110.00	10.41
Pat5	6	3	80	Male	PPMS	None	No	16.99	0.50	0.96	1.75	0.76		1.96	0.51	90.94	31.96
Pat5	6	4	80	Male	PPMS	None	No	20.51	0.50	0.96	0.94	0.74		1.91	0.51	80.67	46.56
Pat5	8	1	80	Male	PPMS	None	No	3.14	0.50	0.96	0.72	0.78	4.43	1.25	0.51	176.20	26.75
Pat5	8	2	80	Male	PPMS	None	No	6.31	0.50	0.96	0.55	0.50	3.66	1.25	0.57	131.68	10.41
Pat5	8	3	80	Male	PPMS	None	No	15.58	0.50	0.96	0.69	0.50	2.40	1.25	0.59	128.96	41.74
Pat5	8	4	40	Male	PPMS	None	No	34.30	0.50	0.96	0.67	0.78	2.17	1.25	0.57	140.87	25.81
Pat5	10	1	80	Male	PPMS	None	No	2.17	0.50	0.96	0.90	0.83	2.19	1.25	0.51	146.66	10.41
Pat5	10	2	80	Male	PPMS	None	No	8.00	0.50	0.96	0.61	0.77	2.55	1.25	0.51	106.55	16.69
Pat5	10	3	80	Male	PPMS	None	No	8.59	0.50	0.96	0.66	0.75	1.39	1.25	0.51	88.44	33.55
Pat5	10	4	80	Male	PPMS	None	No	12.26	0.50	0.96	0.58	0.50	0.94	1.25	0.51	73.63	10.41
Pat6	2	1	40	Male	RRMS	None	No	0.74	21.45	479.94	7.41	2.74		5.98	0.51	141.14	40.38
Pat6	2	2	60	Male	RRMS	None	No	7.15	0.96	64.93	1.53	0.78		2.24	0.51	115.93	59.05
Pat6	2	3	60	Male	RRMS	None	No	19.17	0.50	29.61	1.10	0.50		1.25	0.51	119.77	39.28
Pat6	2	4	60	Male	RRMS	None	No	29.01	0.50	0.96	1.30	0.79		2.05	0.51	119.72	37.63
Pat6	4	1	80	Male	RRMS	None	No	0.50	0.50	3.89	0.76	0.72		1.25	0.51	116.80	10.41
Pat6	4	2	80	Male	RRMS	None	Yes	13.05	0.50	0.96	1.34	0.72		1.91	0.51	102.32	10.41
Pat6	4	3	80	Male	RRMS	None	No	5.41	0.50	1.35	0.97	0.72		2.13	0.51	112.07	33.68
Pat6	4	4	80	Male	RRMS	None	No	8.20	0.50	20.55	0.50	0.69		1.98	0.51	112.78	29.50

Suppl. Table 1B Steroid concentrations in CSF from MS patients after intrathecal TCA administration

Patient	TCA treat-ment cycle	Intra-thecal appli-cation	TCA dose [mg]	Gender	Clinical subtype of MS	BBB dysfu-nction	Intrathecal IgG synthesis	TCA in serum [ng/ml]	TCA in CSF [ng/ml]	Cortico-sterone in CSF [ng/ml]	Cortisol in CSF [ng/ml]	11-Keto-testo-sterone in CSF [ng/ml]	Andro-sten-dione in CSF [ng/ml]	17α,20β-DP in CSF [ng/ml]	Progesterone in CSF [ng/ml]	Estriol in CSF [ng/ml]
Pat1	18	1	80	Male	PPMS	Slight	No	1.64	24.35	0.50	1.65	0.65	0.50	1.25	0.51	10.41
Pat1	18	2	80	Male	PPMS	None	Yes	12.51	1680.23	0.50	0.96	0.50	0.50	1.25	0.51	10.41
Pat1	18	3	80	Male	PPMS	None	Yes	18.81	1761.46	0.50	0.96	0.66	0.50	1.25	0.87	10.41
Pat1	18	4	80	Male	PPMS	None	Yes	36.24	6565.86	1.01	0.96	0.50	0.79	1.25	0.51	10.41
Pat1	19	1	80	Male	PPMS	Slight	No	1.00	0.50	0.50	1.90	0.67	0.50	1.25	0.51	10.41
Pat1	19	2	80	Male	PPMS	Slight	Yes	9.32	3873.36	0.50	0.96	0.50	0.50	1.25	0.51	10.41
Pat1	19	3	80	Male	PPMS	Moderate	No	6.35	3109.44	0.50	0.96	0.66	0.50	1.25	0.87	10.41
Pat1	19	4	80	Male	PPMS	Slight	Yes	20.29	6719.75	0.50	0.96	0.70	0.50	1.25	0.51	10.41
Pat1	20	1	80	Male	PPMS	Slight	No	1.04	90.31	0.50	11.41	0.61	0.71	1.25	0.71	10.41
Pat1	20	2	80	Male	PPMS	None	Yes	8.05	3817.90	0.50	0.96	0.50	0.50	1.25	0.70	10.41
Pat1	20	3	80	Male	PPMS	Slight	No	8.80	5775.93	0.50	0.96	0.64	0.50	1.25	0.51	10.41
Pat1	20	4	80	Male	PPMS	Slight	No	24.39	9912.67	0.50	0.96	0.50	0.72	1.25	0.71	10.41
Pat1	21	1	80	Male	PPMS	Slight	No	3.02	43.03	0.50	16.19	0.61	0.50	1.25	0.69	10.41
Pat1	21	2	80	Male	PPMS	Slight	Yes	6.51	2632.86	0.50	0.96	0.61	0.50	1.25	0.70	10.41
Pat1	21	3	80	Male	PPMS	Slight	No	4.22	4583.01	0.50	0.96	0.63	0.50	1.25	0.51	19.90
Pat1	21	4	40	Male	PPMS	Moderate	No	13.35	7345.06	0.50	0.96	0.50	0.50	1.25	0.69	10.41
Pat2	8	1	40	Female	SPMS	None	No	1.01	0.50	0.50	5.71	0.50	0.78	1.25	0.51	10.41
Pat2	8	2	40	Female	SPMS	Slight	No	6.87	1358.59	0.50	0.96	0.50	0.50	1.25	0.87	10.41
Pat2	8	3	40	Female	SPMS	None	No	7.25	497.69	0.50	0.96	0.50	0.50	1.25	0.51	10.41
Pat2	8	4	40	Female	SPMS	None	No	8.38	1341.82	0.50	0.96	0.50	0.79	1.25	0.51	10.41
Pat2	9	1	40	Female	SPMS	None	No	4.56	8.22	0.50	1.02	0.50	0.50	1.25	0.87	10.41
Pat2	9	2	40	Female	SPMS	None	No	6.31	739.69	0.50	0.96	0.50	0.79	1.25	0.51	10.41
Pat2	9	3	40	Female	SPMS	None	Yes	15.32	1475.05	0.50	0.96	0.50	0.50	1.25	0.88	10.41
Pat2	9	4	40	Female	SPMS	None	Yes	14.71	2617.61	0.50	0.96	0.50	0.79	1.25	0.87	10.41
Pat2	14	1	40	Female	SPMS	Moderate	No	0.98	0.50	0.50	36.35	0.50	0.50	1.25	0.51	10.41
Pat2	14	2	40	Female	SPMS	Slight	No	10.04	2152.01	0.50	0.96	0.50	0.71	1.25	0.51	10.41
Pat2	14	3	40	Female	SPMS	Slight	No	6.85	2807.61	0.50	0.96	0.50	0.71	1.25	0.51	10.41
Pat2	14	4	40	Female	SPMS	Slight	No	8.83	2601.70	0.50	0.96	0.50	0.50	1.25	0.71	10.41

Patient	TCA treat-ment cycle	Intra-thecal appli-cation	TCA dose [mg]	Gender	Clinical subtype of MS	BBB dysfu-nction	Intrathecal IgG synthesis	TCA in serum [ng/ml]	TCA in CSF [ng/ml]	Cortico-sterone in CSF [ng/ml]	Cortisol in CSF [ng/ml]	11-Keto-testo-sterone in CSF [ng/ml]	Andro-sten-dione in CSF [ng/ml]	17α,20β-DP in CSF [ng/ml]	Progesterone in CSF [ng/ml]	Estriol in CSF [ng/ml]
Pat3	4	1	60	Male	SPMS	None	No	6.72	159.65	0.50	1.32	0.76	0.50	1.25	0.80	10.41
Pat3	4	2	60	Male	SPMS	Slight	No	3.80	1497.67	0.50	1.05	0.50	0.78	1.25	0.80	10.41
Pat3	4	3	60	Male	SPMS	Slight	No	6.92	2669.04	0.50	0.96	0.76	0.50	1.25	0.80	10.41
Pat3	4	4	60	Male	SPMS	Moderate	No	5.62	3297.28	0.50	0.96	0.50	0.79	1.25	0.51	10.41
Pat3	5	1	40	Male	SPMS	Slight	No	0.50	325.38	0.94	1.02	0.84	0.79	1.25	0.81	10.41
Pat3	5	2	40	Male	SPMS	Slight	No	2.94	2468.92	0.50	0.96	0.76	0.78	1.25	0.51	10.41
Pat3	5	3	60	Male	SPMS	Moderate	No	5.65	1809.55	0.50	0.96	0.77	0.50	1.25	0.81	10.41
Pat3	5	4	60	Male	SPMS	Moderate	No	5.71	2041.35	0.50	0.96	0.50	0.50	1.25	0.80	10.41
Pat3	10	1	80	Male	SPMS	None	No	1.62	0.50	0.50	28.23	0.50	0.71	1.25	0.70	10.41
Pat3	10	2	80	Male	SPMS	None	No	7.84	3966.45	0.50	0.96	0.62	0.50	1.25	0.70	10.41
Pat3	10	3	80	Male	SPMS	None	No	6.67	1876.59	0.50	0.96	0.50	0.71	1.25	0.51	10.41
Pat3	10	4	40	Male	SPMS	None	No	11.03	2722.39	0.50	0.96	0.63	0.71	1.25	0.72	10.41
Pat3	11	1	60	Male	SPMS	None	No	1.18	40.92	0.50	2.70	0.64	0.50	1.25	0.70	10.41
Pat3	11	2	60	Male	SPMS	None	No	3.58	1516.36	0.50	0.96	0.61	0.50	1.25	0.69	10.41
Pat3	11	3	80	Male	SPMS	None	No	11.70	2466.90	0.50	0.96	0.67	0.71	1.25	0.70	10.41
Pat3	11	4	40	Male	SPMS	None	Yes	21.27	2944.61	0.50	0.96	0.50	0.50	1.25	0.51	10.41
Pat4	2	1	60	Female	SPMS	Moderate	No	0.60	63.15	0.50	0.96	0.50	0.50	1.25	0.51	10.41
Pat4	2	2	60	Female	SPMS	Moderate	No	11.36	4046.45	0.50	0.96	0.50	0.79	1.25	0.51	10.41
Pat4	2	3	60	Female	SPMS	Moderate	No	17.88	7091.76	0.95	0.96	0.50	0.82	1.25	0.84	10.41
Pat4	2	4	60	Female	SPMS	Moderate	No	16.52	7934.31	1.07	0.96	0.50	0.79	1.25	0.51	10.41
Pat4	3	1	60	Female	SPMS	Moderate	No	0.58	277.29	0.50	0.96	0.50	0.50	1.25	0.51	10.41
Pat4	3	2	60	Female	SPMS	Moderate	No	12.68	4554.82	0.50	0.96	0.50	0.79	1.25	0.80	10.41
Pat4	3	3	60	Female	SPMS	Moderate	No	114.54	7470.12	0.50	0.97	0.50	0.79	1.25	0.51	10.41
Pat4	3	4	60	Female	SPMS	Moderate	No	21.51	7002.87	0.50	0.96	0.50	0.79	1.25	0.51	10.41
Pat4	6	1	60	Female	SPMS	Slight	No	1.12	121.66	0.50	33.26	0.50	0.71	1.25	0.51	10.41
Pat4	6	2	60	Female	SPMS	None	No	6.05	3461.53	0.50	0.96	0.50	0.50	1.25	0.51	10.41
Pat4	6	3	60	Female	SPMS	Moderate	No	15.05	8143.33	0.50	0.96	0.50	0.74	1.25	0.51	10.41
Pat4	6	4	60	Female	SPMS	Moderate	No	25.28	8420.66	0.50	0.96	0.50	0.50	1.25	0.51	10.41

Patient	TCA treatment cycle	Intrathecal application	TCA dose [mg]	Gender	Clinical subtype of MS	BBB dysfunction	Intrathecal IgG synthesis	TCA in serum [ng/ml]	TCA in CSF [ng/ml]	Corticosterone in CSF [ng/ml]	Cortisol in CSF [ng/ml]	11-Ketotestosterone in CSF [ng/ml]	Androstendione in CSF [ng/ml]	17α,20β-DP in CSF [ng/ml]	Progesterone in CSF [ng/ml]	Estriol in CSF [ng/ml]
Pat5	5	1	60	Male	PPMS	None	No	0.58	137.54	0.50	0.96	0.50	0.78	1.25	0.82	10.41
Pat5	5	2	60	Male	PPMS	None	No	5.37	266.56	0.92	0.96	0.76	0.78	1.25	0.84	10.41
Pat5	5	3	60	Male	PPMS	None	No	13.78	874.00	0.50	0.96	0.76	0.50	1.25	0.51	10.41
Pat5	5	4	60	Male	PPMS	None	No	7.03	770.89	0.50	0.96	0.50	0.78	1.25	0.81	10.41
Pat5	6	1	80	Male	PPMS	None	No	1.22	110.47	0.50	0.96	0.78	0.50	1.25	0.80	10.41
Pat5	6	2	80	Male	PPMS	None	No	2.48	251.72	0.93	0.96	0.50	0.79	1.25	0.51	10.41
Pat5	6	3	80	Male	PPMS	None	No	16.99	434.13	0.50	0.96	0.50	0.78	1.25	0.51	10.41
Pat5	6	4	80	Male	PPMS	None	No	20.51	801.36	0.50	0.96	0.50	0.50	1.25	0.83	10.41
Pat5	8	1	80	Male	PPMS	None	No	3.14	636.23	0.50	0.96	0.68	0.50	1.25	0.51	10.41
Pat5	8	2	80	Male	PPMS	None	No	6.31	1725.27	0.50	0.96	0.68	0.79	1.25	0.51	10.41
Pat5	8	3	80	Male	PPMS	None	No	15.58	2176.28	0.50	0.96	0.69	0.79	1.25	0.87	10.41
Pat5	8	4	40	Male	PPMS	None	No	34.30	5887.70	0.50	0.96	0.66	0.80	1.25	0.51	10.41
Pat5	10	1	80	Male	PPMS	None	No	2.17	141.00	1.01	0.96	0.50	0.79	1.25	0.87	10.41
Pat5	10	2	80	Male	PPMS	None	No	8.00	425.25	0.50	0.96	0.67	0.50	1.25	0.88	10.41
Pat5	10	3	80	Male	PPMS	None	No	8.59	2203.40	1.04	0.96	0.66	0.50	1.25	0.88	10.41
Pat5	10	4	80	Male	PPMS	None	No	12.26	2861.37	1.00	0.96	0.50	0.50	1.25	0.88	10.41
Pat6	2	1	40	Male	RRMS	None	No	0.74	0.50	0.94	2.66	1.02	0.50	1.25	0.51	10.41
Pat6	2	2	60	Male	RRMS	None	No	7.15	781.65	0.50	0.98	0.80	0.78	1.25	0.81	10.41
Pat6	2	3	60	Male	RRMS	None	No	19.17	288.35	0.92	0.96	0.79	0.50	1.25	0.81	10.41
Pat6	2	4	60	Male	RRMS	None	No	29.01	1687.28	0.94	0.96	0.97	0.78	1.25	0.80	10.41
Pat6	4	1	80	Male	RRMS	None	No	0.50	74.90	0.50	0.96	0.50	0.50	1.25	0.51	10.41
Pat6	4	2	80	Male	RRMS	None	Yes	13.05	1823.37	0.50	0.96	0.80	0.50	1.25	0.51	10.41
Pat6	4	3	80	Male	RRMS	None	No	5.41	126.07	0.50	0.96	0.78	0.78	1.25	0.51	10.41
Pat6	4	4	80	Male	RRMS	None	No	8.20	931.27	0.94	0.96	0.76	0.50	1.25	0.79	10.41
